# A rare case of left atrial appendage aneurysm

**DOI:** 10.1186/s13019-024-02629-7

**Published:** 2024-06-07

**Authors:** Ke Qin, Peng Teng, Liping Shi, Liang Ma

**Affiliations:** Department of Cardiovascular Surgery, The First Affifiliated Hospital, College of Medicine, Zhejiang University, Hangzhou, Zhejiang Province 310003 China

**Keywords:** Congenital left atrial appendage deformity, Left atrial appendage aneurysm

## Abstract

**Background:**

Left atrial appendage aneurysm is a rare cardiac mass, with only a few cases reported. There are usually no specific symptoms, and a few patients visit the doctor with symptoms.

**Case presentation:**

A 20-year-old male presented to our hospital with a “pericardial cyst found by medical evaluation in another hospital for 2 years.” Cardiac ultrasound performed at clinics of our hospital suggested a cystic dark area in the left ventricular lateral wall and the anterior lateral wall, consistent with a pericardial cyst and mild mitral regurgitation. After further relevant examinations and ruling out contraindications, an excision of the left atrial appendage aneurysm was performed under general anesthesia and cardiopulmonary bypass with beating—heart. The postoperative pathological results identified that: (left atrial appendage) fibrocystic wall-like tissue with a focal lining of the flat epithelium, consistent with a benign cyst.

**Conclusion:**

Left atrial appendage aneurysms are rare and insidious. They are usually found by chance during medical evaluations. If the location is not good or the volume is too large, then compression symptoms or arrhythmia, thrombosis and other concomitant symptoms will occur. Surgical resection is presently the only effective radical cure for a left atrial appendage aneurysm.

## Background

Cardiac space-occupying diseases are mostly benign, and mostare myxomas. One of these diseases, the left atrial appendage aneurysm (LAAA), is a rare cardiac mass that can be divided into congenital and acquired types. A congenital left atrial appendage aneurysm is caused by dysplasia of the atrial muscle fibers [1], whereas the acquired type is formed by the gradual expansion of left atrial appendage caused by increased left atrial pressure [2]. In 1982, Foale et al. proposed the following diagnostic criteria of congenital LAAA: (1) It originated from a normal atrium structure; (2) There is a clear communication with the atrial cavity; (3) It is located in the pericardium; (4) The aneurysm causes left ventricular distortion [3]. Williams divides LAAA into two types: extra-pericardial and intra-pericardial [4].

## Case presentation

**Fig. 1:**
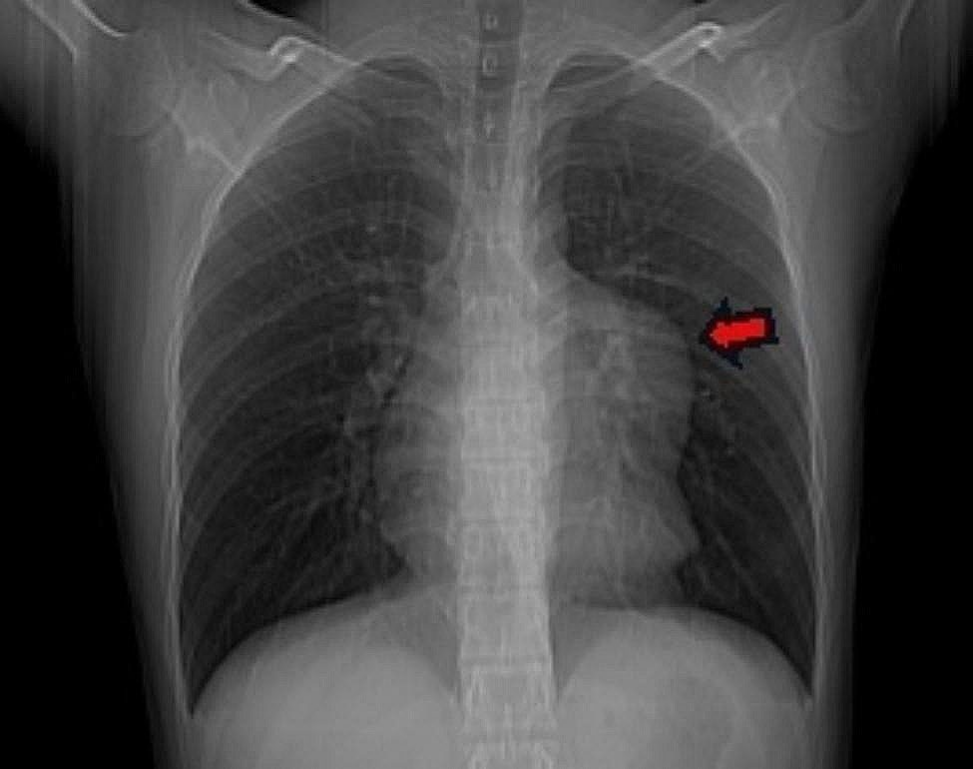
Chest X-ray

**Fig. 2:**
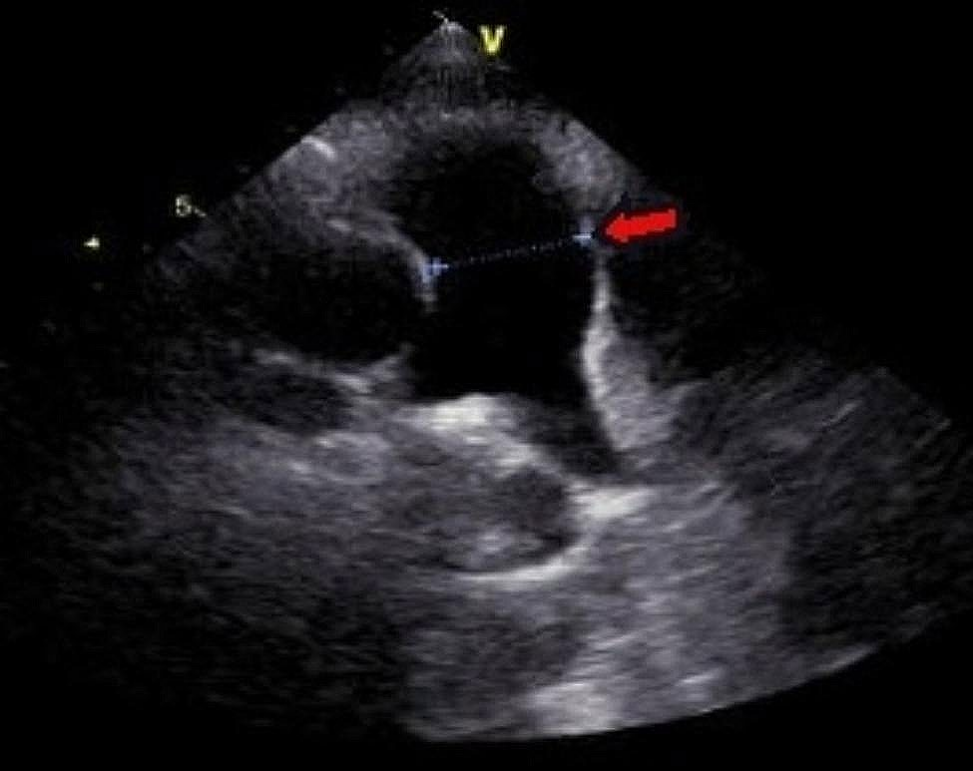
Echocardiography

**Fig. 3:**
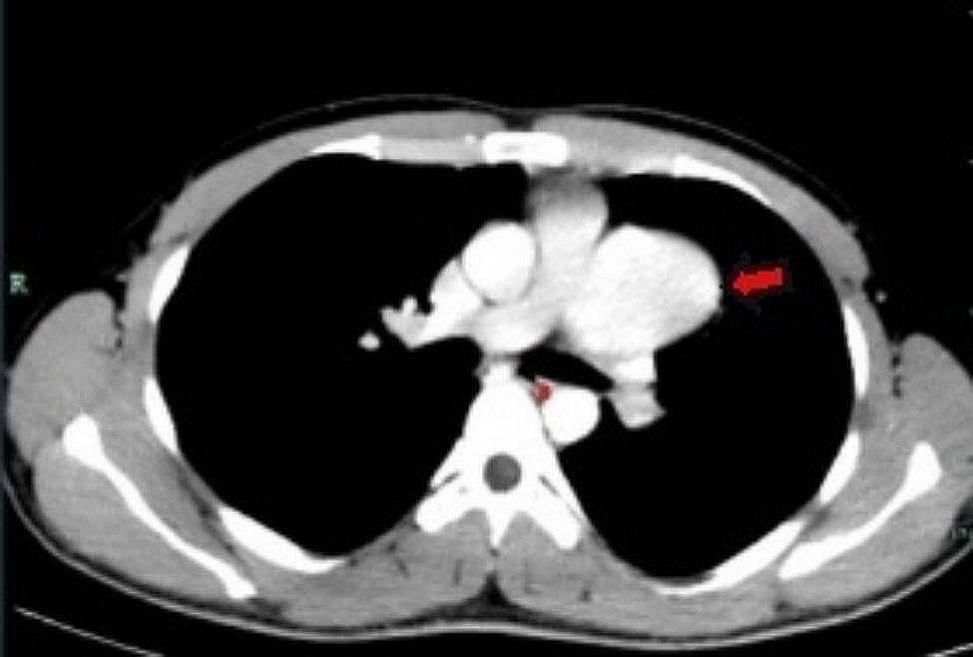
Chest-enhanced computed tomography

**Fig. 4:**
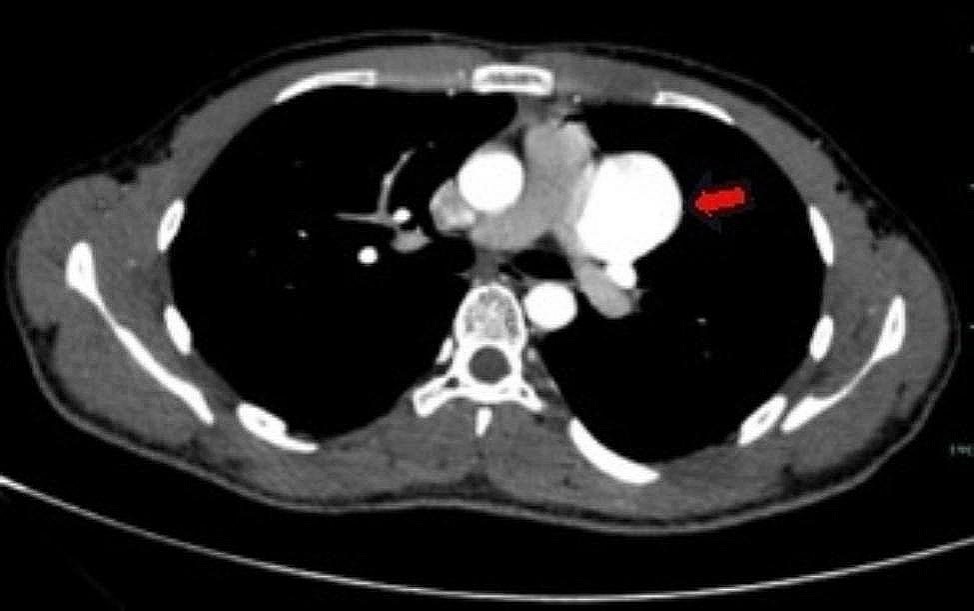
Computed tomography angiography of pulmonary vein

The patient in this case was a 20-year-old male. He was admitted to our hospital with a 2-year history of pericardial cyst. He has no obvious discomfort and displayed no special positive signs. A chest X-ray examination (Fig. 1) showed bulging of the upper segment of the heart. Echocardiographic findings (Fig. 2) included a cystic dark area on the left ventricular lateral wall and anterolateral wall, consistent with a pericardial cyst and mild mitral regurgitation. Chest-enhanced computed tomography (CT) (Fig. 3) showed that the left margin of the heart was saccular and the main pulmonary vein was tumor-like. Computed tomography angiography (CTA) of the pulmonary vein (Fig. 4) indicated a local tumor-like dilatation of the left atrium. No further obvious abnormalities were found in the other laboratory tests and inspection results. Based on the symptoms, signs, auxiliary examinations and medical history analysis, the patient was diagnosed as mild mitral regurgitation, however, because the texture and function of the valve leaflet were good, a secondary left atrial appendage tumor could be ruled out. In addition, the patient’s pericardium was continuous and complete, therefore, he was finally diagnosed as intrapericardial congenital LAAA.

**Fig. 5:**
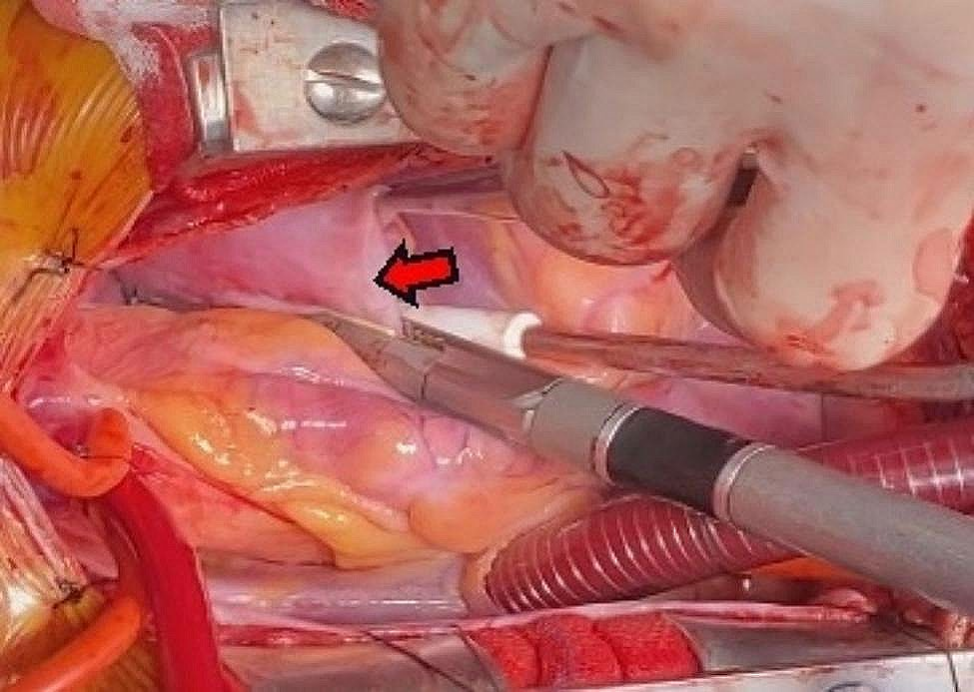
Left atrial appendage aneurysm during the operation

**Fig. 6:**
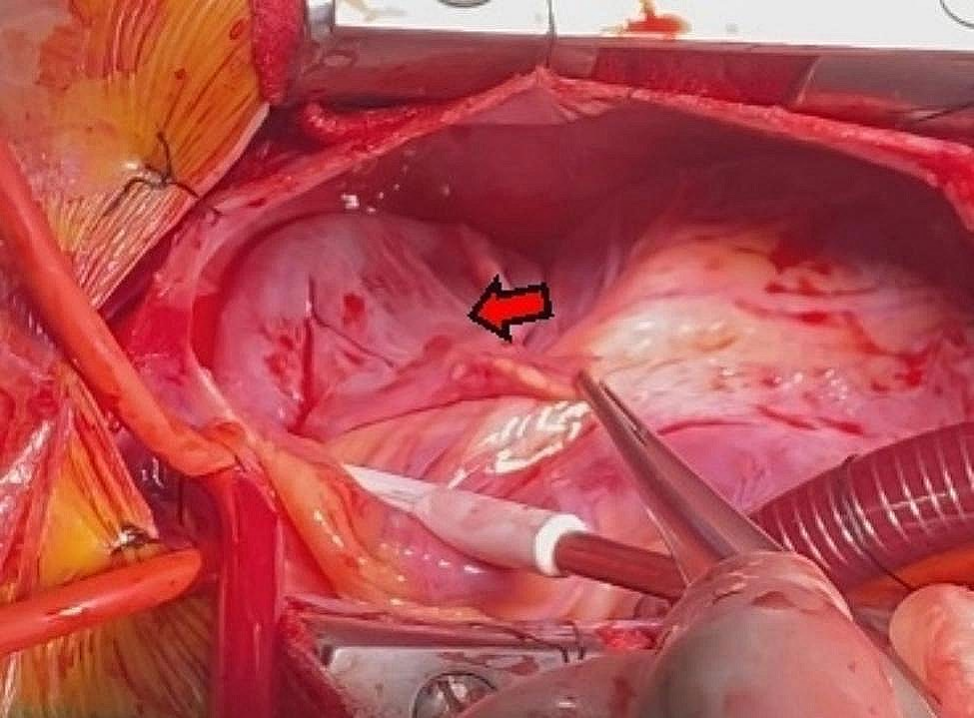
Resecting of left atrial appendage aneurysm

**Fig. 7:**
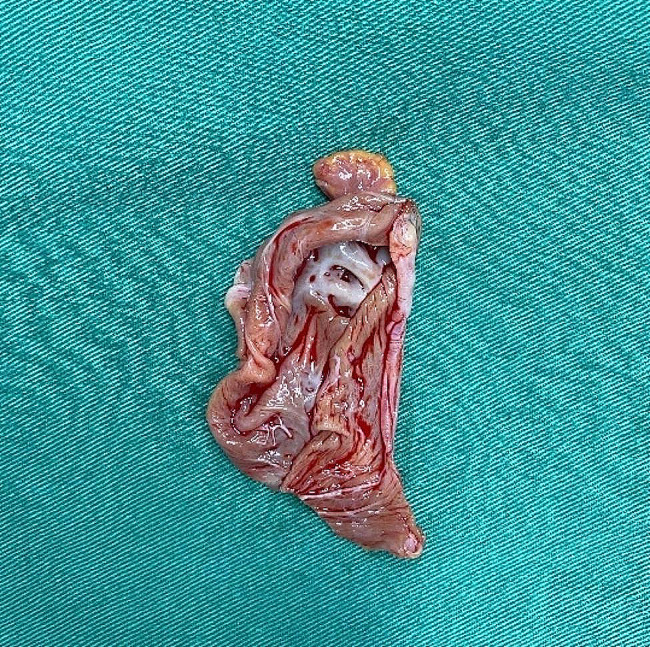
The resected specimens

**Fig. 8:**
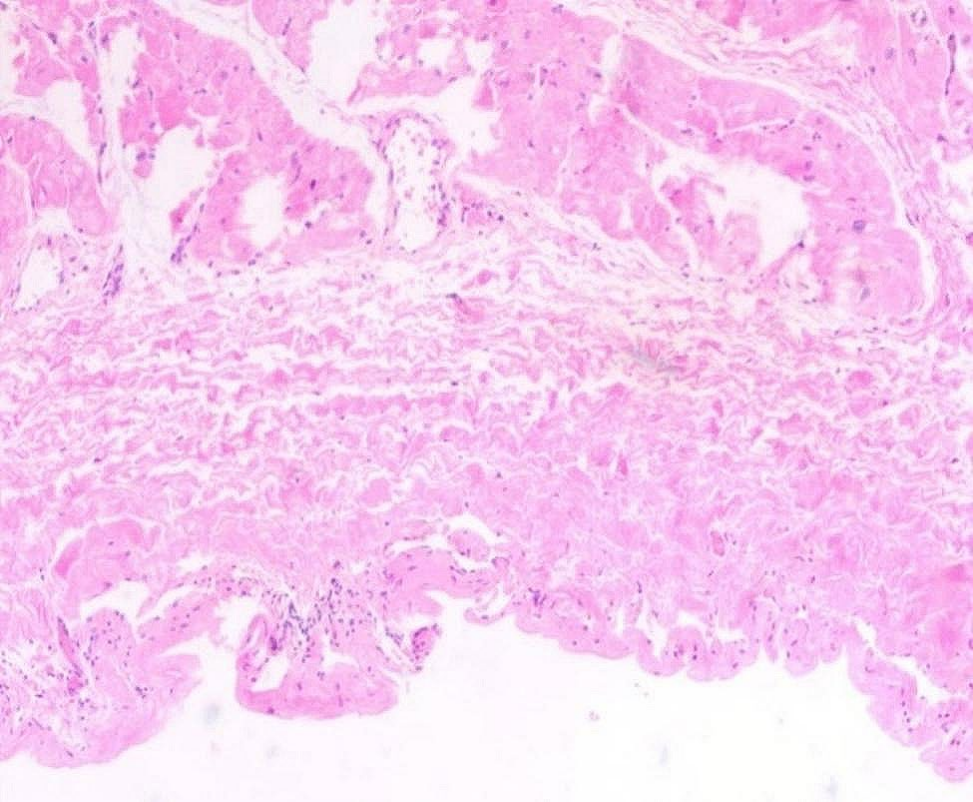
The routine pathology

The treatment was surgical resection. The surgical plan was resection of the atrial appendage aneurysm with beating-heart under general anesthesia and cardiopulmonary bypass. The surgical incision was a median sternotomy. The intubation methods adopted were vena cava intubation and aorta intubation. After opening the pericardium, the left atrial appendage showed tumor-like expansion, measuring about 7 × 7 cm (Fig. 5). The expanded aneurysm was compressing the left upper pulmonary vein and the pulmonary artery trunk; its wall was thin, and the blood flow in it was slow. Upon exploring the atrial appendage aneurysm to its root, we found an obvious pedicle structure, so we chose to cut the aneurysm along the pedicle with a cutting stapler and gold nail (Fig. 6). After confirming that the circumflex branch vessel was not affected and that no no obvious bleeding was present, we performed local reinforcement and suturing. The operation was smoothly completed, as confirmed by intraoperative transesophageal echocardiography. A bedside chest X-ray examination on the same day after the operation suggested the presence of many annular metal shadows in the middle of the chest, and the heart showed postoperative changes, but the rest of the X-ray findings were normal. The patient’s bedside transthoracic echocardiography prompts indicated no obvious abnormal echo mass was found in the left atrial appendage, and hhis heart rate was 132 beats/min. A routine pathological report of the excised left atrial appendage specimen (Fig. 7) showed fibrocystic wall-like tissue with a focal lining of flat epithelium was consistent with a benign cyst (Fig. 8).

The patient recovered smoothly and the tracheal intubation was removed on the first day after the operation. He was transferred back to the general ward the next day and was successfully discharged from the hospital on the sixth day after the operation. Echocardiography before his discharge showed that mitral and tricuspid valves were slightly regurgitated. A coronary CTA showed no obvious abnormal changes in the coronary artery. The blood biochemical examinations showed no abnormality.

The patient returned to the outpatient clinic for re-examination one month after the operation. Echocardiography showed mild tricuspid regurgitation after resection of the LAAA. The re-examination results of the patient six months after the operation yielded results like those described above.

## Discussion

LAAA is a relatively rare cardiac malformation characterized by local or diffuse expansion of the left atrial appendage [5]. At present, only slightly more than 100 related cases of LAAA have been reported. Dimond was the first person to find this disease and reported it in 1960 [6]. The epidemiological characteristics indicate that LAAA has no specific age or gender distribution, and has no special genetic history.

In its pure form, LAAA usually has no specific symptoms. In fact, LAAA is mainly found through an auxiliary examination during a health check-up. A small number of patients are diagnosed in hospital due to particular symptoms, such as palpitation, chest tightness and dyspnea. If the aneurysm is large or poorly located, it will exert pressure on the surrounding tissues or organs, thereby causing dyspnea, angina pectoris, chronic heart failure and other related symptoms. If the aneurysm compresses the left atrium for a prolonged time, this will lead to an increase in pressure in the atrium, the dilatation of the left atrium and other pathophysiological changes, which will then cause the patient to show mitral insufficiency, arrhythmia and other related symptoms. In addition, the blood flow in the aneurysm is often slow, making thrombus formation easier. Once the thrombus dislodges, it will lead to a series of embolism symptoms [1, 7, 8]. In our case, the patient did not show any special symptoms, and the LAAA was accidentally discovered during a routine medical evaluation. Another point worth mentioning is that most patients who have LAAA with atrial fibrillation complications are usually viewed as having large aneurysm and long course of disease. However, Aryal et al. found that the only variable that was significantly related to thrombosis was atrial fibrillation/atrial flutter; other factors, such as patient’s age and gender and the type and size of the LAAA, were not related to thrombosis [1].

The most important auxiliary examination for the diagnosis of LAAA is echocardiography. When compared with transthoracic echocardiography, transesophageal echocardiography can provide a clearer and more accurate comprehension of the internal structure of the heart lesion and allow the determination of whether other organic or functional are involved [9]. Other auxiliary inspections can aid in further understanding the different situations of LAAA when necessary. For example, CT and CTA can intuitively reveal the anatomical structure, relative position and possible compression of an atrial appendage aneurysm. By contrast, MRI and PET-CT are seldom used in clinical cardiac examinations [5]. In our case, the patient’s preoperative examination included two routine examinations: echocardiography and chest X-ray. We also sought to further our understanding of this patient’s LAAA situation, particularly regarding the shape, location and related anatomical relationship of the aneurysm, therefore, we also conducted some targeted examinations, specifically chest CT, chest enhanced CT and pulmonary vascular enhanced CT.

At present, no targeted drugs are available to treat LAAA. However, symptomatic treatment, such as anticoagulants, antiarrhythmic drugs, diuretics and other drugs, can be provided based on the series of complications. Once LAAA is diagnosed, elective resection is recommended. Surgical resection is the radical treatment scheme of LAAA, and it is also the current clinical consensus [5]. Specific surgical schemes include off-pump aneurysm resection, and on-pump aneurysm resection with or without beating heart, among others. The incision approaches include median thoracotomy, right chest mini-incision and total thoracoscopy. At present, the most common treatment choice is aneurysm resection under cardiopulmonary bypass through a median sternotomy approach, and pericardial reconstruction is feasible if necessary [8]. In our case, the patient did not show any accompanying symptoms; therefore, he had no need for targeted drug treatment before and after the operation. Surgical resection was the only and most effective treatment for him. Our preoperative multi-evaluation and intraoperative exploration revealed that the shape of his LAAA was complete, the boundary with the surrounding tissues was clear, and the aneurysm pedicle was obvious. Therefore, the surgeon chose a directly excision of the aneurysm with a cutting stapler under cardiopulmonary bypass with beating heart. During this type of operation, the heart is beating all the time, so going through the processes of cooling/ rewarming and cardiac arrest/ re-beating is unnecessary and obviously shortens the total operation time. To a certain extent, this can also greatly reduce the degree of stress placed on the patients and is conducive to rapid recovery after surgery.

The postoperative prognosis of patients with LAAA is good. To date, no reports of postoperative recurrence, thromboembolism, arrhythmia or other related complications have been reported [1]. In our case, the patient recovered well. He showed no obvious abnormality during the postoperative re-examination or during further re-examinations at 1 month, 6 months and 1 year after discharge.

In summary, as a rare disease, LAAA has the specific characteristics of hidden onset, single treatment and good prognosis. The incidence of LAAA is low, and because its onset is hidden, missing its diagnosis is easy in the clinic. If not detected and treated early and in the absence of sequelae of the disease, LAAA can develop and lead to compression symptoms and even structural changes of the heart, which can lead to heart failure in severe cases. Therefore, we will report this case that was successfully discovered and successfully cured. At the same time, we have also summarized the symptoms and signs, examination methods, diagnosis methods, treatment plans and possible prognosis of LAAA for the reference of our colleagues. However, the research on LAAA is not sufficient at present, and many questions remain. Is there a direct relationship between the size and morphology of LAAA and its complications? What is the standard of surgical treatment for LAAA? Is there any possibility of spontaneous rupture of the aneurysm? No detailed research reports exist that address these issues, and no clinical consensus has yet been reached. In short, further in-depth study is needed on LAAA.

## Conclusion

LAAA is a rare disease and its incidence is hidden. consequently, it is generally found by accidental medical evaluation. If the position is poor or the volume is too large, the patient will show compression symptoms or accompanying symptoms, such as arrhythmia and thrombosis. Echocardiography is the first choice for LAAA, and cardiac CT and MRI can also provide effective help in the treatment of this disease when necessary. At present, the only and effective radical treatment for LAAA is surgical resection.

## Data Availability

Data sharing is not applicable to this article as no datasets were generated or analysed.

## Abbreviations

LAAA: Left atrial appendage aneurysm
